# Evaluating Park Use and Satisfaction: The Case of Trojan Park in St. Louis Missouri

**DOI:** 10.3390/ijerph16152798

**Published:** 2019-08-06

**Authors:** Diana C. Parra, Alexandria Van Zandt, Peter Wang, Micah Goodman, Janardan Abhishek, Debra Haire-Joshu, Ross C. Brownson

**Affiliations:** 1 Program in Physical Therapy, School of Medicine, Washington University in St. Louis, 4444 Forest Park Ave, Campus Box 8502, St. Louis, MO 63108, USA; 2 Department of Pathology and Immunology, School of Medicine, Washington University in St. Louis, St. Louis, MO 63108, USA; 3 Department of Psychiatry, School of Medicine, Washington University in St. Louis, St. Louis, MO 63108, USA; 4 Department of Biology, School of Medicine, Washington University in St. Louis, 1 Brookings Drive, St. Louis, MO 63130, USA; 5 Brown School, Washington University in St. Louis, St. Louis, MO 63130, USA; 6 Center for Diabetes Translation Research, Brown School, Washington University in St. Louis, St. Louis, MO 63130, USA; 7 Prevention Research Center in St. Louis, Brown School at Washington University in St. Louis, 1 Brookings Drive, Campus Box 1196, St. Louis, MO 63130, USA; 8 Department of Surgery (Division of Public Health Sciences) and Alvin J. Siteman Cancer Center, Washington University School of Medicine, Washington University in St. Louis, St. Louis, MO 63130, USA

**Keywords:** parks, fitness zones, SOPARC, physical activity, multigenerational playground

## Abstract

*Background*: Providing public access to exercise and play is vital for health promotion across populations. We evaluated the use of and satisfaction at Trojan Park, a multigenerational playground with multiple activity areas and fitness zones in the city of Wellston in St. Louis County, MO. *Methods*: We used video footage and the System for Observing Play and Recreation in Communities (SOPARC), which is a valid and reliable system for collecting data on physical activity in parks. We then performed intercept interviews to gather user information and measure overall satisfaction with the park. *Results*: The park received a variety of attendees across age groups, with children and middle-aged adults representing 41.1% and 50.3% of total park users, respectively. During the time observed, 47% of attendees were engaged in moderate to vigorous physical activity (MVPA), 22% were engaged in light physical activity (walking), and 30% were sedentary. We also observed participants spending the most time on the basketball court (38%), playground (28%), and picnic (17%) areas. Park users traveled a wide range of distances to access the park and the overwhelming majority reported a high level of satisfaction. *Conclusions*: Our findings demonstrate that multigenerational playgrounds with access to various activities and fitness zones may provide social and physical health benefits.

## 1. Introduction

Parks are a free resource for the community, providing facilities for physical activities such as basketball courts, soccer fields, walking paths, play structures and, most recently, fitness zones or outdoor fitness equipment. Physical activity improves physical and emotional health, and prevents chronic non-communicable diseases such as diabetes, obesity, and metabolic syndrome. Furthermore, there is strong evidence that physical activity (PA) reduces the rates of all-cause mortality, high blood pressure, stroke, metabolic syndrome, breast cancer, colon cancer, and depression [1]. Conversely, physical inactivity is a strong predictor of obesity [2], diabetes [3], and cardiovascular disease [4]. 

The Center for Disease Control and Prevention (CDC) reports that the prevalence of childhood obesity is 18.5%, affecting 13.7 million children in the United States [5]. The prevalence of obesity in adults is 39.8%, affecting 93.3 million adults in the United States. Lee et al. concluded that physical inactivity is globally responsible for 6% to 10% of the major non-communicable diseases, specifically coronary heart disease, type 2 diabetes, and breast and colon cancers [1]. Additionally, the cost of inactivity is a growing problem in the United States: Carlson et al. found that physical inactivity contributed to 11.1% of aggregate health care expenditures in the US [6]. Ding et al. estimated that physical inactivity costs healthcare systems $53.8 billion dollars worldwide [7]. The research clearly supports the proposition that PA enhances health while physical inactivity contributes to the burden of disease.

According to the Physical Activity Guidelines for Americans 2nd edition released in 2018, adults should undertake at least 150 min to 300 min a week of moderate PA, or 75 min to 150 min a week of vigorous PA, or an equivalent combination of moderate and vigorous physical activity (MVPA) [8]. Children and adolescents aged 6 through 17 years should participate in 60 min or more of MVPA daily [8]. The latest information from 2008 to 2016 reveals that among American adults, only 26% of men, 19% of women, and 20% of adolescents report sufficient activity to meet the relevant aerobic and muscle-strengthening guidelines [8]. In Missouri, the 2014 CDC state report revealed that only 27.2% of youths met the aerobic activity guidelines, and 49.5% achieved at least 150 min a week of moderate physical activity [9]. Thus, promoting physical activity should remain a public health priority.

Though parks provide free opportunities for children and adults to engage in physical exercise, many are underutilized [10], which may be due to several factors such as the neighborhood’s condition, a reduced sense of safety, or the park’s lack of cleanliness [11]. Furthermore, certain park activities typically appeal to youths rather than adults and the elderly [11]. Local communities then face the challenge of developing urban park infrastructures that appeal to a wide age group, while also targeting inactive individuals. Urban parks have traditionally provided openly accessible recreational opportunities to community members; the impact on physical health and activity levels depends on multiple factors including their perceived benefits, the equipment available, accessibility, safety, cleanliness, and personal preferences. Introducing ways to assess these factors would not only help determine the impact of individual parks, but also identify features that could be improved or more widely implemented. 

Trojan Park has implemented Fitness zones, which are free outdoor fitness equipment stations placed in local parks—one of the newer strategies to promote MVPA for a variety of ages [12]. Many countries in Asia, Europe, South America and Australia have already implemented fitness zones in parks [13,14,15,16,17,18]. Cohen et al. define fitness zones as “easy to use outdoor gyms consisting of durable, weather-, and vandal resistant exercise equipment for strength training and aerobic exercise [12].” Fitness zones can also act as multigenerational playgrounds in the sense that they provide an area for adult PA along with playgrounds and other park areas for children. Though there is limited research on fitness zones, they have been shown to increase PA in parks and benefit segments of the population that are at a higher risk of physical inactivity, including women, older adults, children, and individuals who live in low socioeconomic status (SES) neighborhoods [12,14]. Moreover, by virtue of being free to use, fitness zones and accompanying programs are appealing to a segment of the population that could not otherwise afford “for fee” activities such as gym memberships or yoga studios. 

Trojan Park was sponsored by the National Recreation and Park Association’s (NPRA) 2016 Parks Build Community initiative, which partners with communities to augment the value of parks and recreational centers by building new parks and renovating pre-existing parks. Trojan Park is one of the few green spaces in Wellston, MO. Wellston is a city in St. Louis County, Missouri, along the northwest border of the city of St. Louis. Wellston has a population of 2313 according to the latest census, a median household income of approximately $20,423, and a median age of 31 years [19]. The park opened on 8 October, 2016 to provide its residents a place to play and exercise. Wellston also has a poverty rate of 43.5%, which is much higher than the national average of 13.4%. Furthermore, only 79.3% of the population has healthcare coverage. The population of Wellston is 96.4% Black, 1.98% White, and 1.65% two or more races [19].

We used Trojan Park as a case study to investigate park use, satisfaction and the opinions of park users regarding improvements to parks. We measured park use by conducting park intercept surveys and analyzing video surveillance footage, combined with the SOPARC method. We obtained permission for the video surveillance from Great Rivers Greenway [20], which is a non-profit public organization that supports the development of local parks of St. Louis and has Trojan park under its management. The intercept surveys identified park user demographics, exercise preferences, safety concerns and their opinions of Trojan Park concerning potential improvements that would enhance their park experience. This data will be valuable in adding to the literature of urban park use and satisfaction, as well as the use of fitness zones.

## 2. Materials and Methods 

### 2.1. System for Observing Play and Recreation in Communities (SOPARC)

**Figure 1:**
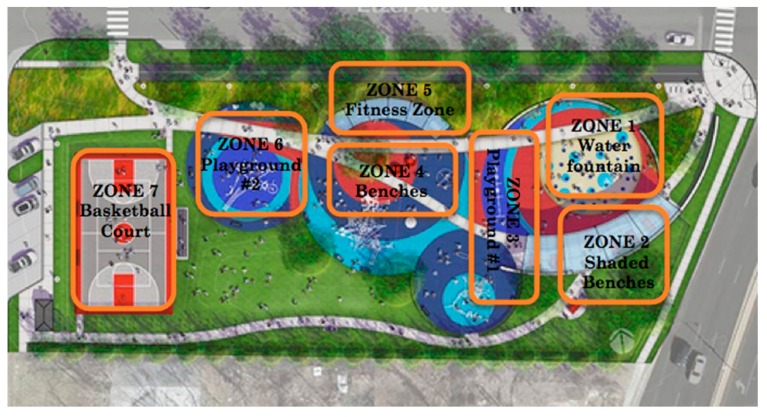
Map of Trojan Park divided into seven zones for scanning.

We used the System for Observing Play and Recreation in Communities (SOPARC) to observe and collect data. The SOPARC has been shown to be reliable, valid, and useful in recording the physical activities seen in various parks [21]. Furthermore, the SOPARC is a useful tool for showing relationships between park characteristics, park user characteristics, levels of PA, and relative levels of use among the park’s different areas. The SOPARC method divides the park into specific boundaries, which are then periodically observed for park user characteristics and their type of PA [22]. We divided Trojan park into seven sequential zones that were scanned (Figure 1). Using the live video footage provided by Great Rivers Greenway, we used the SOPARC method to count and classify park users according to age, gender, race and physical activity level. We scanned these specific areas four times per day for an hour each (08:00, 11:00, 19:00, and 21:00) on alternating days, over two weeks in July 2018—including both weekends and weekdays—for a total of 13 days of observation. This was based on previously established criteria on how many days of observation are enough to obtain reliable estimates through the SOPARC [23].

The SOPARC was developed to obtain direct information about the PA level of community public spaces, based on momentary time sampling to make systematic observations (scans) of target-areas. PA was coded as sedentary (lying down, sitting or standing), walking (light walking or moving) or vigorous (moderate or vigorous walking, running, strength activities). Age groups were divided into children, adolescents, adults and older adults. Observations were performed by two trained observers under the supervision of one field coordinator. The training was conducted during a 2-day workshop and included classes designed to familiarize trainees with operational definitions, instrument notation, coding conventions and the categorization of PA levels and age groups. Trainees practiced coding and received feedback on their scoring. After the field training, the two observers carried out two days of observation through the video, to test for inter-observer agreement. Ninety percent agreement was obtained for both PA levels and age group categories. The validity of the activity codes used by the SOPARC has been established through heart rate monitoring [24].

### 2.2. Intercept Interviews

In addition, we conducted park intercept surveys of park users over a period of approximately four weeks in July 2018, at 19:00. for approximately one hour on alternating days. We used a modified version of a park intercept survey previously used by Cohen et al. which has been shown to demonstrate high measures of reliability and validity [25,26,27] (See Appendix A). Only adults who completed an oral informed consent form and were over 18 years old were interviewed. The Institutional Review Board’s (IRB) approval for this study (#201805037) was obtained from Washington University in St. Louis prior to beginning data collection.

### 2.3. Data Analysis

We performed statistical analysis to describe contextual and users’ characteristics using descriptive and non-parametric statistics. Users’ characteristics (gender, age group, race, and PA level) were analyzed by target zone. Users’ characteristics were also analyzed by days of the week and period (time) of the day. All comparisons were tested using *chi-square* to test for statistically significant differences in STATA software version 13 (Stata 13 Base Reference Manual. College Station, TX, USA). For the intercept interviews, we used REDCAP software (Washington University in St. Louis, St. Louis, MO, USA) to enter the data and produced data descriptive characteristics reports using this software. 

## 3. Results

### 3.1. Live Video Feed Using the SOPARC

Over 13 days and a total of 52 observations, 599 people visited the park, averaging about 46 people per day. There were significant differences in the number of visitors on weekends versus weekdays, as well as in the time of the day, age, gender, and race (Table 1). The majority of people visited from 19:00 to 20:00 and were Black, males, and either children or adults. Nearly half of the visitors engaged in MVPA and the basketball court was the most-used area. Of the 599 total visitors, only 9% used the fitness zone, of whom the vast majority were children. The results are presented in Table 1.

### 3.2. Intercept Interviews

In addition to using the SOPARC, we interviewed 93 individuals asking them questions pertaining to their personal usage of, satisfaction with, and feelings of safety in Trojan Park—each interview lasted approximately 20 min. We approached 100 people and 93 agreed to participate—a response rate of 93%. Of the respondents, 44% were male, and 56% were female, and the majority were Black (92%), 6% were Hispanic/Latino, and 3% were White. We noted significant differences in race and disability status (Table 2). 

The survey revealed that the vast majority of respondents were “very satisfied” with the park. Furthermore, all of the participants reported feeling “very safe” or “safe”. Most of the respondents stated that they used the park a few times a week (34%) and reported that the park was very easy (84%) and easy (15%) to get to. The most common reason to visit the park was taking children to the playground (39%), which was followed closely by using the basketball court (37%), sitting in the park (28%), meeting friends, (27%), and using the fitness zone (26%). The majority of the participants from the survey stated that they used the park for exercise (42%), more so than going to a gym (31%) or exercising at home (26%). In terms of the frequency of visits, 34.1% stated they visit the park a few times a week and 13% reported they come to Trojan Park daily. The survey also asked participants to select all the features they would most like to see in the park. The most popular of the options included seeing more park events/fairs and competitions (57.9%), park concerts/dances (52.6%), youth sports leagues (51.3%), adult sports leagues, (46.1%), and fitness classes such as aerobics and Zumba (38.2%). All of the participants said that they would like to see more parks like Trojan Park in St. Louis (Table 3).

## 4. Discussion

Improving access to shared spaces for enrichment and play is an important part of public health. However, little is known about the factors influencing the usage and perceived impact of such places. Particularly in the U.S. urban setting, these are significant questions with potentially far-reaching implications—especially for populations of low socioeconomic status and ethnic minorities. Furthermore, recent research shows that parks in low socioeconomic and ethnic minority neighborhoods have lower quality parks with reduced levels of safety, compared to parks in higher socioeconomic, predominantly White neighborhoods [28]. Trojan Park is located in a low income and predominantly Black county and is therefore a valuable case study for evaluating park satisfaction, safety and use in a low socioeconomic and ethnic minority neighborhood. 

The intercept survey revealed that the majority of park users were Black, as 92% of the respondents identified as being Black, compared to 5.7% who identified as Hispanic/Latino, and 3.4% as White. This corresponded with the results from the systematic observation obtained with the use of the SOPARC. As of 2017, Wellston’s racial demographics were composed of approximately 96% Black, 2% White, and 2% of 2 or more races. The percentages obtained through the use of the SOPARC in this study, as well as the intercept surveys, are very similar to the percentages of the overall county, which indicate that the demographics of the park users were representative of the surrounding population [29]. The majority of respondents were females (56%), which is different from the systematic observation results where we observed 35% females; this is probably due to the different times that the observations were done compared to the intercept interview, or possibly to a higher likelihood of women answering the survey compared to men. The African American or Black population, specifically African American women, have some of the highest rates of physical inactivity in the country [30]. Trojan Park is located in a low-income county of St. Louis, which is a high-risk population for physical inactivity [31]. Though we did not ask respondents their socioeconomic status, Wellston has a high poverty rate of 44% and the majority of park users stated that they lived less than one (40%) or two (19%) miles away from the park and most of the respondents (83.5%) stated that the park was very easy to get to. Other studies reported in the literature have found similar results, including a four-city study that showed that park users were mostly likely to reside within 0.5 and 1 mile and thus be representative of the surrounding community [26]. Trojan Park is an important resource for promoting physical activity in populations at risk of physical inactivity, specifically Black and low-income individuals. Results from the intercept survey also revealed that 47% of the respondents visited the park on a daily basis or a few times a week. In addition, the most common length of stay at the park was between 1 and 2 h (37%), followed by between 2 and 5 h (47%), which further supports the potential of the park to facilitate and increase PA among the surrounding community. In fact, 20% of the respondents were visiting the park for the first time on the day of the survey, and 29% had visited only during the last 6 months. This indicates a great potential for reaching new populations and for the park attracting new users every day, with a great potential for the sustainability and maintenance of physical activity practice. 

This study sought to gain a better insight into how to enhance and build future parks that appeal to park users. The survey data revealed that 42% of respondents usually go to the park for exercise, which shows the importance of maximizing park infrastructure to facilitate physical activity. Similar results have been published in the literature assessing fitness zones, which have found that the placement of such infrastructure or of organized activities increases park use [14,15,17]. Such infrastructure has been implemented within the park in zones near playgrounds, where adults can potentially use the resources while the children play. Most importantly however, besides increasing park use, these studies have documented an increase in the level of vigorous PA—particularly among groups traditionally at risk of inactivity such as women and older adults [14,15,17]. The strength and resistance training equipment included with the fitness zones’ infrastructure facilitates engagement in MVPA. At Trojan park, taking kids to play followed by using the basketball courts, sitting in the park and fitness zones were the most commonly reported activities in which respondents participated when at the park, according to the intercept interviews. The systematic observation showed similar results, with the exception of fitness zones, where 26% of the respondents stated they usually used the equipment—a finding not supported by our observational data using the SOPARC, which showed that this zone was only used 9% of the time and mostly by children (73%). The majority of the users rated their confidence as high (a mean of 8.5, where 10 is the most confident) for using the fitness equipment, which implies that fitness zones are user-friendly to the general population, but there is still a disconnect between their perception and their actual use. Previous research supports that fitness zones in parks may indeed be a cost-effective way to help individuals engage in more physical activity, especially MVPA [12]. Community programs and strategies that can engage residents in using this equipment could be a valuable intervention, particularly among women, due to the proximity of the fitness zones to the playground. Caretakers, especially mothers, could engage in MVPA using the fitness zones while children use the playground. The intercept survey revealed that 39% of the respondents reported “Taking kids to play” as the reason why they visited the park, and 49% reported visiting the park with the family group, further supporting the potential of Trojan park to be a multigenerational playground. 

In regards to implementing park activities, most respondents desired to have park events or fairs, concerts, and dances, followed by youth and adult sports leagues. This is supported by prior research showing that organized and supervised activities are stronger predictors of increased park use than targeting perceived threats such as crime and the presence of a homeless population [31]. Regarding park infrastructure improvement, the top three desired features included better exercise equipment, pull up bars and punching bags. Implementing these changes and introducing park organized activities may further maximize the park’s potential in promoting physical activity. Our results support the findings from Cohen et al., which showed that neighborhood poverty levels, the perception of safety and the presence of incivilities were not associated with the number of park users observed. On the other hand, organized activities and the number of activity facilities were strongly correlated with park use [26].

This study has important strengths and limitations, which should be noted. First, the use of combined self-reported and observational data increases the comprehensiveness of our results and acts as a triangulation method to be able to compare and validate results. By focusing on a low-income population, we were able to better understand the value of public infrastructure in high-risk areas. An important limitation was the collection of data during one season (only during July 2018), where the average temperature during the two weeks of data collection was 84 °F. This could have affected our results, as people may decide to increase or decrease usage depending on the daily temperature. As a result, our results are not generalizable to other seasons and months of the year. 

## 5. Conclusions

The intercept survey data reveals valuable insights regarding park users’ preferences and opinions of Trojan park, which will help further the development of parks that appeal to populations at risk of physical inactivity. All of the respondents were either very satisfied or satisfied with Trojan Park and 100% desired to see more parks like this in St. Louis, which supports the proposition that Trojan park may function as a good template for building other urban parks. Furthermore, all respondents felt safe or very safe in Trojan park. Safety and park accessibility are major components of park use [31], which may contribute to this park being successful. 

While playground access in the U.S. has generally been limited to children, Trojan Park offers built-in features that cater to multiple generations. Parks facilitate physical activity, and our results support the importance of parks for physical activity in a variety of age groups. Our findings indicate that multigenerational playgrounds provide an important source of physical activity and social engagement for community members. In addition to fitness zones, playgrounds and basketball courts also served as preferred recreational facilities for children and adults, respectively. Indeed, multigenerational accessibility may be a key component to a more widespread adoption of healthy practices at urban parks. Future studies may expand on ways to enhance the impact of new and existing playgrounds by increasing access across age groups, through equipment design or the implementation of organized activities. Our results suggest that complementing traditional playground equipment with additional features such as fitness zones, family picnic areas, basketball courts and organized activities may be a useful strategy for increasing park visitation, usage, and overall satisfaction. These have important implications for health equity and community engagement, particularly in underserved populations.

## Figures and Tables

**Table 1:** Results from the systematic observation and video footage.

	Count	Percentage	*Chi-Square*
Period			
Weekdays	231	39%	30.8 *
Weekend days	368	61%	
Time			
08:00–09:00	9	2%	527.7 *
11:00–12:00	52	9%	
19:00–20:00	372	62%	
21:00–22:00	166	28%	
Age			
Children	246	41%	426.2 *
Adolescents	49	8%	
Adults	301	50%	
Older Adults	3	1%	
Gender			
Female	207	35%	56.5 *
Male	392	65%	
Race			
Black	584	97%	1109.7 *
White	6	1%	
Hispanic	9	2%	
Activity level			
Sedentary	183	31%	56.9 *
Walking	134	22%	
Vigorous	282	47%	
Zone			
Benches	8	1%	340.6 *
Water Fountain	42	7%	
Fitness Zones	56	9%	
Playground with swings	70	12%	
Playground	93	16%	
Shaded picnic tables	104	17%	
Basketball Court	226	38%	

* Significant at the 0.001 *p*-value.

**Table 2:** Park User Demographic characteristics based on intercept interviews.

Park User Characteristics	Count	Percentage	*Chi-Square*
Park User Distance			18.4 *
1/4 Mile Resident	10	11%	
1/2 Mile Resident	11	12%	
1 Mile Resident	15	17%	
2 Mile Resident	17	19%	
Other Distance	32	36%	
Gender			
Female	50	56%	
Male	39	44%	1.1
Race			
Black	80	92%	128.2 *
White	3	3%	
Latino	5	6%	
Disability Status			
Physical Disability	3	3%	74.5 *
No Physical Disability	85	97%	

* Significant at the 0.001 *p*-value.

**Table 3:** Park user opinions based on intercept interview results.

Selected Intercept Survey Questions	Count	Percentage
Level of Satisfaction		
Very satisfied	83	90%
Somewhat satisfied	9	10%
Level or sense of safety		
Very safe	59	66%
Safe	30	34%
How often do you come to this Park		
Daily/A few times a week	43	47%
1× per week/A couple times per month	24	26%
Monthly/A few times a year	8	8%
This is the first time	16	18%
Length of time spent at the Park		
15–60 min	11	12%
More than 1 h but less than 2	34	37%
Between 2 and 5 h	43	47%
More than 5 h	4	4%
First time you came to the Park		
Today	18	20%
In the past 6 months	27	29%
Between 6 months and 2 years	40	44%
More than 2 years ago	6	6.6%
With Whom do you visit the park		
Alone	10	11%
Family Group	45	49%
Spouse/Children	50	54%
Friends/Organized Group/Nanny or babysitter	39	42%
Desired park activities		
Adult sports leagues	35	46%
Adult dance classes	19	25%
Fitness classes	29	38%
Youth sports leagues	39	51%
Organized adventure/walks	15	20%
Park events/fairs, competitions	44	58%
Park concerts/dances	40	53%
Yoga	13	17%
How easy is it for you to get to the park		
Very easy	76	84%
Easy	14	15%
How do you usually get to the park		
Walk	24	26%
Bike	2	2%
Bus or other public transportation	7	8%
Other (car)	71	78%
What do you usually do at the park		
Fitness Zone (outdoor exercise equipment)	24	26%
Basketball	34	37%
Celebrations, picnics	1	1%
Soccer	11	12%
Meet Friends	25	27%
Playground	16	17%
Water Fountain	14	15%
Meet new people	21	23%
Sitting in park (relax)	26	28%
Skating	16	17%
Taking kids to play	36	39%
Walking/Jogging	2	2%
Walking with dog	2	2%
Confidence in the ability to use the outdoor fitness equipment		
Scale from 1 to 10, with 10 being extremely confident (Mean/Standard deviation)	8.5	2.2
Where do you usually exercise?		
Park	36	42%
Home	22	26%
Private health club/Gym/YMCA	34	39%
Outdoors/other	18	21%
I don’t usually exercise	11	13%

* Significant at the 0.001 *p*-value.

**Figure A1:**
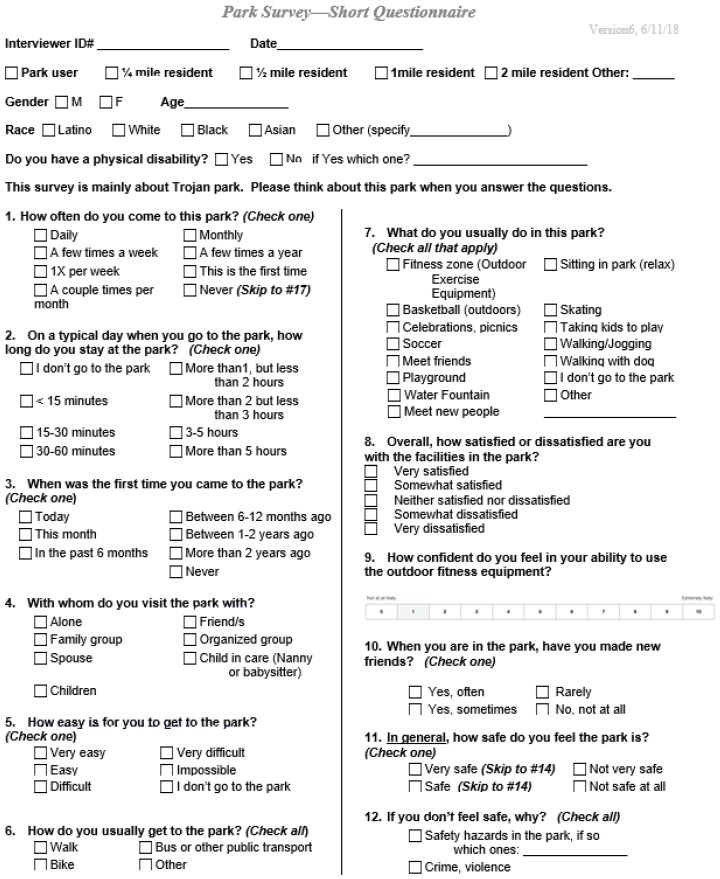

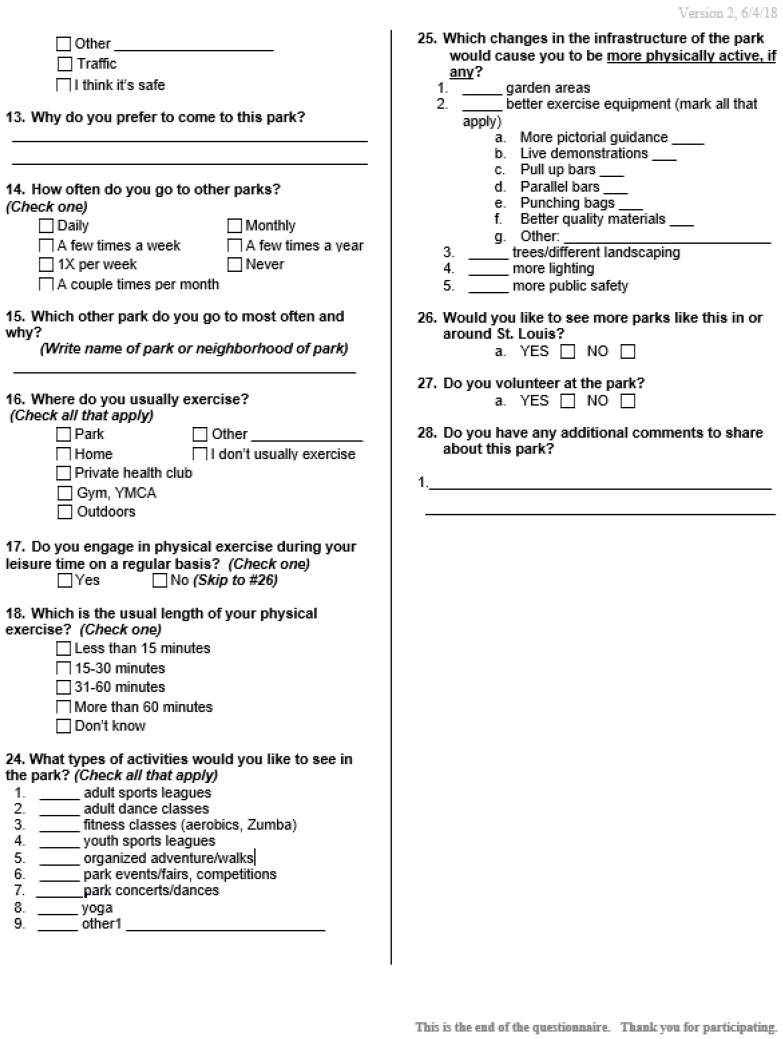
Park Intercept Survey.
